# Supplementary material for: The effects of genital myiasis on the diversity of the vaginal microbiota in female Bactrian camels
Source: BMC Vet Res. 2022 Mar 5;18:87. doi: 10.1186/s12917-022-03189-5 (PMC8897907; doi:10.1186/s12917-022-03189-5)
Supplement: Supplementary file 5 — Additional file 5. [file 12917_2022_3189_MOESM5_ESM.zip › MPL201709200_16s_yy/Treat1/css/js_css/jsPDF-master.zip!/jsPDF-master/docs/jspdf.js.html]

jspdf.js - Documentation


## Home

### Classes

- jsPDF

### Global

- addFont
- addHTML
- addMetadata
- addPage
- autoPrint
- CapJoinStyles
- circle
- ellipse
- getFontList
- lines
- lstext
- output
- rect
- roundedRect
- save
- setDisplayMode
- setDrawColor
- setFillColor
- setFont
- setFontSize
- setFontStyle
- setLineCap
- setLineJoin
- setLineWidth
- setPage
- setProperties
- setTextColor
- text
- triangle

# jspdf.js

```
/** @preserve
 * jsPDF - PDF Document creation from JavaScript
 * Version ${versionID}
 *                           CommitID ${commitID}
 *
 * Copyright (c) 2010-2016 James Hall <james@parall.ax>, https://github.com/MrRio/jsPDF
 *               2010 Aaron Spike, https://github.com/acspike
 *               2012 Willow Systems Corporation, willow-systems.com
 *               2012 Pablo Hess, https://github.com/pablohess
 *               2012 Florian Jenett, https://github.com/fjenett
 *               2013 Warren Weckesser, https://github.com/warrenweckesser
 *               2013 Youssef Beddad, https://github.com/lifof
 *               2013 Lee Driscoll, https://github.com/lsdriscoll
 *               2013 Stefan Slonevskiy, https://github.com/stefslon
 *               2013 Jeremy Morel, https://github.com/jmorel
 *               2013 Christoph Hartmann, https://github.com/chris-rock
 *               2014 Juan Pablo Gaviria, https://github.com/juanpgaviria
 *               2014 James Makes, https://github.com/dollaruw
 *               2014 Diego Casorran, https://github.com/diegocr
 *               2014 Steven Spungin, https://github.com/Flamenco
 *               2014 Kenneth Glassey, https://github.com/Gavvers
 *
 * Permission is hereby granted, free of charge, to any person obtaining
 * a copy of this software and associated documentation files (the
 * "Software"), to deal in the Software without restriction, including
 * without limitation the rights to use, copy, modify, merge, publish,
 * distribute, sublicense, and/or sell copies of the Software, and to
 * permit persons to whom the Software is furnished to do so, subject to
 * the following conditions:
 *
 * The above copyright notice and this permission notice shall be
 * included in all copies or substantial portions of the Software.
 *
 * THE SOFTWARE IS PROVIDED "AS IS", WITHOUT WARRANTY OF ANY KIND,
 * EXPRESS OR IMPLIED, INCLUDING BUT NOT LIMITED TO THE WARRANTIES OF
 * MERCHANTABILITY, FITNESS FOR A PARTICULAR PURPOSE AND
 * NONINFRINGEMENT. IN NO EVENT SHALL THE AUTHORS OR COPYRIGHT HOLDERS BE
 * LIABLE FOR ANY CLAIM, DAMAGES OR OTHER LIABILITY, WHETHER IN AN ACTION
 * OF CONTRACT, TORT OR OTHERWISE, ARISING FROM, OUT OF OR IN CONNECTION
 * WITH THE SOFTWARE OR THE USE OR OTHER DEALINGS IN THE SOFTWARE.
 *
 * Contributor(s):
 *    siefkenj, ahwolf, rickygu, Midnith, saintclair, eaparango,
 *    kim3er, mfo, alnorth, Flamenco
 */

/**
 * Creates new jsPDF document object instance.
 *
 * @class
 * @param orientation One of "portrait" or "landscape" (or shortcuts "p" (Default), "l")
 * @param unit        Measurement unit to be used when coordinates are specified.
 *                    One of "pt" (points), "mm" (Default), "cm", "in"
 * @param format      One of 'pageFormats' as shown below, default: a4
 * @returns {jsPDF}
 * @name jsPDF
 */
var jsPDF = (function(global) {
  'use strict';
  var pdfVersion = '1.3',
    pageFormats = { // Size in pt of various paper formats
      'a0': [2383.94, 3370.39],
      'a1': [1683.78, 2383.94],
      'a2': [1190.55, 1683.78],
      'a3': [841.89, 1190.55],
      'a4': [595.28, 841.89],
      'a5': [419.53, 595.28],
      'a6': [297.64, 419.53],
      'a7': [209.76, 297.64],
      'a8': [147.40, 209.76],
      'a9': [104.88, 147.40],
      'a10': [73.70, 104.88],
      'b0': [2834.65, 4008.19],
      'b1': [2004.09, 2834.65],
      'b2': [1417.32, 2004.09],
      'b3': [1000.63, 1417.32],
      'b4': [708.66, 1000.63],
      'b5': [498.90, 708.66],
      'b6': [354.33, 498.90],
      'b7': [249.45, 354.33],
      'b8': [175.75, 249.45],
      'b9': [124.72, 175.75],
      'b10': [87.87, 124.72],
      'c0': [2599.37, 3676.54],
      'c1': [1836.85, 2599.37],
      'c2': [1298.27, 1836.85],
      'c3': [918.43, 1298.27],
      'c4': [649.13, 918.43],
      'c5': [459.21, 649.13],
      'c6': [323.15, 459.21],
      'c7': [229.61, 323.15],
      'c8': [161.57, 229.61],
      'c9': [113.39, 161.57],
      'c10': [79.37, 113.39],
      'dl': [311.81, 623.62],
      'letter': [612, 792],
      'government-letter': [576, 756],
      'legal': [612, 1008],
      'junior-legal': [576, 360],
      'ledger': [1224, 792],
      'tabloid': [792, 1224],
      'credit-card': [153, 243]
    };

  /**
   * jsPDF's Internal PubSub Implementation.
   * See mrrio.github.io/jsPDF/doc/symbols/PubSub.html
   * Backward compatible rewritten on 2014 by
   * Diego Casorran, https://github.com/diegocr
   *
   * @class
   * @name PubSub
   * @ignore This should not be in the public docs.
   */
  function PubSub(context) {
    var topics = {};

    this.subscribe = function(topic, callback, once) {
      if (typeof callback !== 'function') {
        return false;
      }

      if (!topics.hasOwnProperty(topic)) {
        topics[topic] = {};
      }

      var id = Math.random().toString(35);
      topics[topic][id] = [callback, !!once];

      return id;
    };

    this.unsubscribe = function(token) {
      for (var topic in topics) {
        if (topics[topic][token]) {
          delete topics[topic][token];
          return true;
        }
      }
      return false;
    };

    this.publish = function(topic) {
      if (topics.hasOwnProperty(topic)) {
        var args = Array.prototype.slice.call(arguments, 1),
          idr = [];

        for (var id in topics[topic]) {
          var sub = topics[topic][id];
          try {
            sub[0].apply(context, args);
          } catch (ex) {
            if (global.console) {
              console.error('jsPDF PubSub Error', ex.message, ex);
            }
          }
          if (sub[1]) idr.push(id);
        }
        if (idr.length) idr.forEach(this.unsubscribe);
      }
    };
  }

  /**
   * @constructor
   * @private
   */
  function jsPDF(orientation, unit, format, compressPdf) {
    var options = {};

    if (typeof orientation === 'object') {
      options = orientation;

      orientation = options.orientation;
      unit = options.unit || unit;
      format = options.format || format;
      compressPdf = options.compress || options.compressPdf || compressPdf;
    }

    // Default options
    unit = unit || 'mm';
    format = format || 'a4';
    orientation = ('' + (orientation || 'P')).toLowerCase();

    var format_as_string = ('' + format).toLowerCase(),
      compress = !!compressPdf && typeof Uint8Array === 'function',
      textColor = options.textColor || '0 g',
      drawColor = options.drawColor || '0 G',
      activeFontSize = options.fontSize || 16,
      lineHeightProportion = options.lineHeight || 1.15,
      lineWidth = options.lineWidth || 0.200025, // 2mm
      objectNumber = 2, // 'n' Current object number
      outToPages = !1, // switches where out() prints. outToPages true = push to pages obj. outToPages false = doc builder content
      offsets = [], // List of offsets. Activated and reset by buildDocument(). Pupulated by various calls buildDocument makes.
      fonts = {}, // collection of font objects, where key is fontKey - a dynamically created label for a given font.
      fontmap = {}, // mapping structure fontName > fontStyle > font key - performance layer. See addFont()
      activeFontKey, // will be string representing the KEY of the font as combination of fontName + fontStyle
      k, // Scale factor
      tmp,
      page = 0,
      currentPage,
      pages = [],
      pagesContext = [], // same index as pages and pagedim
      pagedim = [],
      content = [],
      additionalObjects = [],
      lineCapID = 0,
      lineJoinID = 0,
      content_length = 0,
      pageWidth,
      pageHeight,
      pageMode,
      zoomMode,
      layoutMode,
      documentProperties = {
        'title': '',
        'subject': '',
        'author': '',
        'keywords': '',
        'creator': ''
      },
      API = {},
      events = new PubSub(API),

      /////////////////////
      // Private functions
      /////////////////////
      f2 = function(number) {
        return number.toFixed(2); // Ie, %.2f
      },
      f3 = function(number) {
        return number.toFixed(3); // Ie, %.3f
      },
      padd2 = function(number) {
        return ('0' + parseInt(number)).slice(-2);
      },
      out = function(string) {
        if (outToPages) {
          /* set by beginPage */
          pages[currentPage].push(string);
        } else {
          // +1 for '\n' that will be used to join 'content'
          content_length += string.length + 1;
          content.push(string);
        }
      },
      newObject = function() {
        // Begin a new object
        objectNumber++;
        offsets[objectNumber] = content_length;
        out(objectNumber + ' 0 obj');
        return objectNumber;
      },
      // Does not output the object until after the pages have been output.
      // Returns an object containing the objectId and content.
      // All pages have been added so the object ID can be estimated to start right after.
      // This does not modify the current objectNumber;  It must be updated after the newObjects are output.
      newAdditionalObject = function() {
        var objId = pages.length * 2 + 1;
        objId += additionalObjects.length;
        var obj = {
          objId: objId,
          content: ''
        };
        additionalObjects.push(obj);
        return obj;
      },
      // Does not output the object.  The caller must call newObjectDeferredBegin(oid) before outputing any data
      newObjectDeferred = function() {
        objectNumber++;
        offsets[objectNumber] = function() {
          return content_length;
        };
        return objectNumber;
      },
      newObjectDeferredBegin = function(oid) {
        offsets[oid] = content_length;
      },
      putStream = function(str) {
        out('stream');
        out(str);
        out('endstream');
      },
      putPages = function() {
        var n, p, arr, i, deflater, adler32, adler32cs, wPt, hPt,
          pageObjectNumbers = [];

        adler32cs = global.adler32cs || jsPDF.adler32cs;
        if (compress && typeof adler32cs === 'undefined') {
          compress = false;
        }

        // outToPages = false as set in endDocument(). out() writes to content.

        for (n = 1; n <= page; n++) {
          pageObjectNumbers.push(newObject());
          wPt = (pageWidth = pagedim[n].width) * k;
          hPt = (pageHeight = pagedim[n].height) * k;
          out('<</Type /Page');
          out('/Parent 1 0 R');
          out('/Resources 2 0 R');
          out('/MediaBox [0 0 ' + f2(wPt) + ' ' + f2(hPt) + ']');
          // Added for annotation plugin
          events.publish('putPage', {
            pageNumber: n,
            page: pages[n]
          });
          out('/Contents ' + (objectNumber + 1) + ' 0 R');
          out('>>');
          out('endobj');

          // Page content
          p = pages[n].join('\n');
          newObject();
          if (compress) {
            arr = [];
            i = p.length;
            while (i--) {
              arr[i] = p.charCodeAt(i);
            }
            adler32 = adler32cs.from(p);
            deflater = new Deflater(6);
            deflater.append(new Uint8Array(arr));
            p = deflater.flush();
            arr = new Uint8Array(p.length + 6);
            arr.set(new Uint8Array([120, 156])),
              arr.set(p, 2);
            arr.set(new Uint8Array([adler32 & 0xFF, (adler32 >> 8) & 0xFF, (
                adler32 >> 16) & 0xFF, (adler32 >> 24) & 0xFF]), p.length +
              2);
            p = String.fromCharCode.apply(null, arr);
            out('<</Length ' + p.length + ' /Filter [/FlateDecode]>>');
          } else {
            out('<</Length ' + p.length + '>>');
          }
          putStream(p);
          out('endobj');
        }
        offsets[1] = content_length;
        out('1 0 obj');
        out('<</Type /Pages');
        var kids = '/Kids [';
        for (i = 0; i < page; i++) {
          kids += pageObjectNumbers[i] + ' 0 R ';
        }
        out(kids + ']');
        out('/Count ' + page);
        out('>>');
        out('endobj');
        events.publish('postPutPages');
      },
      putFont = function(font) {
        font.objectNumber = newObject();
        out('<</BaseFont/' + font.PostScriptName + '/Type/Font');
        if (typeof font.encoding === 'string') {
          out('/Encoding/' + font.encoding);
        }
        out('/Subtype/Type1>>');
        out('endobj');
      },
      putFonts = function() {
        for (var fontKey in fonts) {
          if (fonts.hasOwnProperty(fontKey)) {
            putFont(fonts[fontKey]);
          }
        }
      },
      putXobjectDict = function() {
        // Loop through images, or other data objects
        events.publish('putXobjectDict');
      },
      putResourceDictionary = function() {
        out('/ProcSet [/PDF /Text /ImageB /ImageC /ImageI]');
        out('/Font <<');

        // Do this for each font, the '1' bit is the index of the font
        for (var fontKey in fonts) {
          if (fonts.hasOwnProperty(fontKey)) {
            out('/' + fontKey + ' ' + fonts[fontKey].objectNumber + ' 0 R');
          }
        }
        out('>>');
        out('/XObject <<');
        putXobjectDict();
        out('>>');
      },
      putResources = function() {
        putFonts();
        events.publish('putResources');
        // Resource dictionary
        offsets[2] = content_length;
        out('2 0 obj');
        out('<<');
        putResourceDictionary();
        out('>>');
        out('endobj');
        events.publish('postPutResources');
      },
      putAdditionalObjects = function() {
        events.publish('putAdditionalObjects');
        for (var i = 0; i < additionalObjects.length; i++) {
          var obj = additionalObjects[i];
          offsets[obj.objId] = content_length;
          out(obj.objId + ' 0 obj');
          out(obj.content);;
          out('endobj');
        }
        objectNumber += additionalObjects.length;
        events.publish('postPutAdditionalObjects');
      },
      addToFontDictionary = function(fontKey, fontName, fontStyle) {
        // this is mapping structure for quick font key lookup.
        // returns the KEY of the font (ex: "F1") for a given
        // pair of font name and type (ex: "Arial". "Italic")
        if (!fontmap.hasOwnProperty(fontName)) {
          fontmap[fontName] = {};
        }
        fontmap[fontName][fontStyle] = fontKey;
      },
      /**
       * FontObject describes a particular font as member of an instnace of jsPDF
       *
       * It's a collection of properties like 'id' (to be used in PDF stream),
       * 'fontName' (font's family name), 'fontStyle' (font's style variant label)
       *
       * @class
       * @public
       * @property id {String} PDF-document-instance-specific label assinged to the font.
       * @property PostScriptName {String} PDF specification full name for the font
       * @property encoding {Object} Encoding_name-to-Font_metrics_object mapping.
       * @name FontObject
       * @ignore This should not be in the public docs.
       */
      addFont = function(PostScriptName, fontName, fontStyle, encoding) {
        var fontKey = 'F' + (Object.keys(fonts).length + 1).toString(10),
          // This is FontObject
          font = fonts[fontKey] = {
            'id': fontKey,
            'PostScriptName': PostScriptName,
            'fontName': fontName,
            'fontStyle': fontStyle,
            'encoding': encoding,
            'metadata': {}
          };
        addToFontDictionary(fontKey, fontName, fontStyle);
        events.publish('addFont', font);

        return fontKey;
      },
      addFonts = function() {

        var HELVETICA = "helvetica",
          TIMES = "times",
          COURIER = "courier",
          NORMAL = "normal",
          BOLD = "bold",
          ITALIC = "italic",
          BOLD_ITALIC = "bolditalic",
          encoding = 'StandardEncoding',
          ZAPF = "zapfdingbats",
          standardFonts = [
            ['Helvetica', HELVETICA, NORMAL],
            ['Helvetica-Bold', HELVETICA, BOLD],
            ['Helvetica-Oblique', HELVETICA, ITALIC],
            ['Helvetica-BoldOblique', HELVETICA, BOLD_ITALIC],
            ['Courier', COURIER, NORMAL],
            ['Courier-Bold', COURIER, BOLD],
            ['Courier-Oblique', COURIER, ITALIC],
            ['Courier-BoldOblique', COURIER, BOLD_ITALIC],
            ['Times-Roman', TIMES, NORMAL],
            ['Times-Bold', TIMES, BOLD],
            ['Times-Italic', TIMES, ITALIC],
            ['Times-BoldItalic', TIMES, BOLD_ITALIC],
            ['ZapfDingbats', ZAPF]
          ];

        for (var i = 0, l = standardFonts.length; i < l; i++) {
          var fontKey = addFont(
            standardFonts[i][0],
            standardFonts[i][1],
            standardFonts[i][2],
            encoding);

          // adding aliases for standard fonts, this time matching the capitalization
          var parts = standardFonts[i][0].split('-');
          addToFontDictionary(fontKey, parts[0], parts[1] || '');
        }
        events.publish('addFonts', {
          fonts: fonts,
          dictionary: fontmap
        });
      },
      SAFE = function __safeCall(fn) {
        fn.foo = function __safeCallWrapper() {
          try {
            return fn.apply(this, arguments);
          } catch (e) {
            var stack = e.stack || '';
            if (~stack.indexOf(' at ')) stack = stack.split(" at ")[1];
            var m = "Error in function " + stack.split("\n")[0].split('<')[
              0] + ": " + e.message;
            if (global.console) {
              global.console.error(m, e);
              if (global.alert) alert(m);
            } else {
              throw new Error(m);
            }
          }
        };
        fn.foo.bar = fn;
        return fn.foo;
      },
      to8bitStream = function(text, flags) {
        /**
         * PDF 1.3 spec:
         * "For text strings encoded in Unicode, the first two bytes must be 254 followed by
         * 255, representing the Unicode byte order marker, U+FEFF. (This sequence conflicts
         * with the PDFDocEncoding character sequence thorn ydieresis, which is unlikely
         * to be a meaningful beginning of a word or phrase.) The remainder of the
         * string consists of Unicode character codes, according to the UTF-16 encoding
         * specified in the Unicode standard, version 2.0. Commonly used Unicode values
         * are represented as 2 bytes per character, with the high-order byte appearing first
         * in the string."
         *
         * In other words, if there are chars in a string with char code above 255, we
         * recode the string to UCS2 BE - string doubles in length and BOM is prepended.
         *
         * HOWEVER!
         * Actual *content* (body) text (as opposed to strings used in document properties etc)
         * does NOT expect BOM. There, it is treated as a literal GID (Glyph ID)
         *
         * Because of Adobe's focus on "you subset your fonts!" you are not supposed to have
         * a font that maps directly Unicode (UCS2 / UTF16BE) code to font GID, but you could
         * fudge it with "Identity-H" encoding and custom CIDtoGID map that mimics Unicode
         * code page. There, however, all characters in the stream are treated as GIDs,
         * including BOM, which is the reason we need to skip BOM in content text (i.e. that
         * that is tied to a font).
         *
         * To signal this "special" PDFEscape / to8bitStream handling mode,
         * API.text() function sets (unless you overwrite it with manual values
         * given to API.text(.., flags) )
         * flags.autoencode = true
         * flags.noBOM = true
         *
         * ===================================================================================
         * `flags` properties relied upon:
         *   .sourceEncoding = string with encoding label.
         *                     "Unicode" by default. = encoding of the incoming text.
         *                     pass some non-existing encoding name
         *                     (ex: 'Do not touch my strings! I know what I am doing.')
         *                     to make encoding code skip the encoding step.
         *   .outputEncoding = Either valid PDF encoding name
         *                     (must be supported by jsPDF font metrics, otherwise no encoding)
         *                     or a JS object, where key = sourceCharCode, value = outputCharCode
         *                     missing keys will be treated as: sourceCharCode === outputCharCode
         *   .noBOM
         *       See comment higher above for explanation for why this is important
         *   .autoencode
         *       See comment higher above for explanation for why this is important
         */

        var i, l, sourceEncoding, encodingBlock, outputEncoding, newtext,
          isUnicode, ch, bch;

        flags = flags || {};
        sourceEncoding = flags.sourceEncoding || 'Unicode';
        outputEncoding = flags.outputEncoding;

        // This 'encoding' section relies on font metrics format
        // attached to font objects by, among others,
        // "Willow Systems' standard_font_metrics plugin"
        // see jspdf.plugin.standard_font_metrics.js for format
        // of the font.metadata.encoding Object.
        // It should be something like
        //   .encoding = {'codePages':['WinANSI....'], 'WinANSI...':{code:code, ...}}
        //   .widths = {0:width, code:width, ..., 'fof':divisor}
        //   .kerning = {code:{previous_char_code:shift, ..., 'fof':-divisor},...}
        if ((flags.autoencode || outputEncoding) &&
          fonts[activeFontKey].metadata &&
          fonts[activeFontKey].metadata[sourceEncoding] &&
          fonts[activeFontKey].metadata[sourceEncoding].encoding) {
          encodingBlock = fonts[activeFontKey].metadata[sourceEncoding].encoding;

          // each font has default encoding. Some have it clearly defined.
          if (!outputEncoding && fonts[activeFontKey].encoding) {
            outputEncoding = fonts[activeFontKey].encoding;
          }

          // Hmmm, the above did not work? Let's try again, in different place.
          if (!outputEncoding && encodingBlock.codePages) {
            outputEncoding = encodingBlock.codePages[0]; // let's say, first one is the default
          }

          if (typeof outputEncoding === 'string') {
            outputEncoding = encodingBlock[outputEncoding];
          }
          // we want output encoding to be a JS Object, where
          // key = sourceEncoding's character code and
          // value = outputEncoding's character code.
          if (outputEncoding) {
            isUnicode = false;
            newtext = [];
            for (i = 0, l = text.length; i < l; i++) {
              ch = outputEncoding[text.charCodeAt(i)];
              if (ch) {
                newtext.push(
                  String.fromCharCode(ch));
              } else {
                newtext.push(
                  text[i]);
              }

              // since we are looping over chars anyway, might as well
              // check for residual unicodeness
              if (newtext[i].charCodeAt(0) >> 8) {
                /* more than 255 */
                isUnicode = true;
              }
            }
            text = newtext.join('');
          }
        }

        i = text.length;
        // isUnicode may be set to false above. Hence the triple-equal to undefined
        while (isUnicode === undefined && i !== 0) {
          if (text.charCodeAt(i - 1) >> 8) {
            /* more than 255 */
            isUnicode = true;
          }
          i--;
        }
        if (!isUnicode) {
          return text;
        }

        newtext = flags.noBOM ? [] : [254, 255];
        for (i = 0, l = text.length; i < l; i++) {
          ch = text.charCodeAt(i);
          bch = ch >> 8; // divide by 256
          if (bch >> 8) {
            /* something left after dividing by 256 second time */
            throw new Error("Character at position " + i + " of string '" +
              text + "' exceeds 16bits. Cannot be encoded into UCS-2 BE");
          }
          newtext.push(bch);
          newtext.push(ch - (bch << 8));
        }
        return String.fromCharCode.apply(undefined, newtext);
      },
      pdfEscape = function(text, flags) {
        /**
         * Replace '/', '(', and ')' with pdf-safe versions
         *
         * Doing to8bitStream does NOT make this PDF display unicode text. For that
         * we also need to reference a unicode font and embed it - royal pain in the rear.
         *
         * There is still a benefit to to8bitStream - PDF simply cannot handle 16bit chars,
         * which JavaScript Strings are happy to provide. So, while we still cannot display
         * 2-byte characters property, at least CONDITIONALLY converting (entire string containing)
         * 16bit chars to (USC-2-BE) 2-bytes per char + BOM streams we ensure that entire PDF
         * is still parseable.
         * This will allow immediate support for unicode in document properties strings.
         */
        return to8bitStream(text, flags).replace(/\\/g, '\\\\').replace(
          /\(/g, '\\(').replace(/\)/g, '\\)');
      },
      putInfo = function() {
        out('/Producer (jsPDF ' + jsPDF.version + ')');
        for (var key in documentProperties) {
          if (documentProperties.hasOwnProperty(key) && documentProperties[
              key]) {
            out('/' + key.substr(0, 1).toUpperCase() + key.substr(1) + ' (' +
              pdfEscape(documentProperties[key]) + ')');
          }
        }
        var created = new Date(),
          tzoffset = created.getTimezoneOffset(),
          tzsign = tzoffset < 0 ? '+' : '-',
          tzhour = Math.floor(Math.abs(tzoffset / 60)),
          tzmin = Math.abs(tzoffset % 60),
          tzstr = [tzsign, padd2(tzhour), "'", padd2(tzmin), "'"].join('');
        out(['/CreationDate (D:',
          created.getFullYear(),
          padd2(created.getMonth() + 1),
          padd2(created.getDate()),
          padd2(created.getHours()),
          padd2(created.getMinutes()),
          padd2(created.getSeconds()), tzstr, ')'
        ].join(''));
      },
      putCatalog = function() {
        out('/Type /Catalog');
        out('/Pages 1 0 R');
        // PDF13ref Section 7.2.1
        if (!zoomMode) zoomMode = 'fullwidth';
        switch (zoomMode) {
          case 'fullwidth':
            out('/OpenAction [3 0 R /FitH null]');
            break;
          case 'fullheight':
            out('/OpenAction [3 0 R /FitV null]');
            break;
          case 'fullpage':
            out('/OpenAction [3 0 R /Fit]');
            break;
          case 'original':
            out('/OpenAction [3 0 R /XYZ null null 1]');
            break;
          default:
            var pcn = '' + zoomMode;
            if (pcn.substr(pcn.length - 1) === '%')
              zoomMode = parseInt(zoomMode) / 100;
            if (typeof zoomMode === 'number') {
              out('/OpenAction [3 0 R /XYZ null null ' + f2(zoomMode) + ']');
            }
        }
        if (!layoutMode) layoutMode = 'continuous';
        switch (layoutMode) {
          case 'continuous':
            out('/PageLayout /OneColumn');
            break;
          case 'single':
            out('/PageLayout /SinglePage');
            break;
          case 'two':
          case 'twoleft':
            out('/PageLayout /TwoColumnLeft');
            break;
          case 'tworight':
            out('/PageLayout /TwoColumnRight');
            break;
        }
        if (pageMode) {
          /**
           * A name object specifying how the document should be displayed when opened:
           * UseNone      : Neither document outline nor thumbnail images visible -- DEFAULT
           * UseOutlines  : Document outline visible
           * UseThumbs    : Thumbnail images visible
           * FullScreen   : Full-screen mode, with no menu bar, window controls, or any other window visible
           */
          out('/PageMode /' + pageMode);
        }
        events.publish('putCatalog');
      },
      putTrailer = function() {
        out('/Size ' + (objectNumber + 1));
        out('/Root ' + objectNumber + ' 0 R');
        out('/Info ' + (objectNumber - 1) + ' 0 R');
      },
      beginPage = function(width, height) {
        // Dimensions are stored as user units and converted to points on output
        var orientation = typeof height === 'string' && height.toLowerCase();
        if (typeof width === 'string') {
          var format = width.toLowerCase();
          if (pageFormats.hasOwnProperty(format)) {
            width = pageFormats[format][0] / k;
            height = pageFormats[format][1] / k;
          }
        }
        if (Array.isArray(width)) {
          height = width[1];
          width = width[0];
        }
        if (orientation) {
          switch (orientation.substr(0, 1)) {
            case 'l':
              if (height > width) orientation = 's';
              break;
            case 'p':
              if (width > height) orientation = 's';
              break;
          }
          if (orientation === 's') {
            tmp = width;
            width = height;
            height = tmp;
          }
        }
        outToPages = true;
        pages[++page] = [];
        pagedim[page] = {
          width: Number(width) || pageWidth,
          height: Number(height) || pageHeight
        };
        pagesContext[page] = {};
        _setPage(page);
      },
      _addPage = function() {
        beginPage.apply(this, arguments);
        // Set line width
        out(f2(lineWidth * k) + ' w');
        // Set draw color
        out(drawColor);
        // resurrecting non-default line caps, joins
        if (lineCapID !== 0) {
          out(lineCapID + ' J');
        }
        if (lineJoinID !== 0) {
          out(lineJoinID + ' j');
        }
        events.publish('addPage', {
          pageNumber: page
        });
      },
      _deletePage = function(n) {
        if (n > 0 && n <= page) {
          pages.splice(n, 1);
          pagedim.splice(n, 1);
          page--;
          if (currentPage > page) {
            currentPage = page;
          }
          this.setPage(currentPage);
        }
      },
      _setPage = function(n) {
        if (n > 0 && n <= page) {
          currentPage = n;
          pageWidth = pagedim[n].width;
          pageHeight = pagedim[n].height;
        }
      },
      /**
       * Returns a document-specific font key - a label assigned to a
       * font name + font type combination at the time the font was added
       * to the font inventory.
       *
       * Font key is used as label for the desired font for a block of text
       * to be added to the PDF document stream.
       * @private
       * @function
       * @param fontName {String} can be undefined on "falthy" to indicate "use current"
       * @param fontStyle {String} can be undefined on "falthy" to indicate "use current"
       * @returns {String} Font key.
       */
      getFont = function(fontName, fontStyle) {
        var key;

        fontName = fontName !== undefined ? fontName : fonts[activeFontKey]
          .fontName;
        fontStyle = fontStyle !== undefined ? fontStyle : fonts[
          activeFontKey].fontStyle;

        if (fontName !== undefined) {
          fontName = fontName.toLowerCase();
        }
        switch (fontName) {
          case 'sans-serif':
          case 'verdana':
          case 'arial':
          case 'helvetica':
            fontName = 'helvetica';
            break;
          case 'fixed':
          case 'monospace':
          case 'terminal':
          case 'courier':
            fontName = 'courier';
            break;
          case 'serif':
          case 'cursive':
          case 'fantasy':
          default:
            fontName = 'times';
            break;
        }

        try {
          // get a string like 'F3' - the KEY corresponding tot he font + type combination.
          key = fontmap[fontName][fontStyle];
        } catch (e) {}

        if (!key) {
          //throw new Error("Unable to look up font label for font '" + fontName + "', '"
          //+ fontStyle + "'. Refer to getFontList() for available fonts.");
          key = fontmap['times'][fontStyle];
          if (key == null) {
            key = fontmap['times']['normal'];
          }
        }
        return key;
      },
      buildDocument = function() {
        outToPages = false; // switches out() to content

        objectNumber = 2;
        content_length = 0;
        content = [];
        offsets = [];
        additionalObjects = [];
        // Added for AcroForm
        events.publish('buildDocument');

        // putHeader()
        out('%PDF-' + pdfVersion);

        putPages();

        // Must happen after putPages
        // Modifies current object Id
        putAdditionalObjects();

        putResources();

        // Info
        newObject();
        out('<<');
        putInfo();
        out('>>');
        out('endobj');

        // Catalog
        newObject();
        out('<<');
        putCatalog();
        out('>>');
        out('endobj');

        // Cross-ref
        var o = content_length,
          i, p = "0000000000";
        out('xref');
        out('0 ' + (objectNumber + 1));
        out(p + ' 65535 f ');
        for (i = 1; i <= objectNumber; i++) {
          var offset = offsets[i];
          if (typeof offset === 'function') {
            out((p + offsets[i]()).slice(-10) + ' 00000 n ');
          } else {
            out((p + offsets[i]).slice(-10) + ' 00000 n ');
          }
        }
        // Trailer
        out('trailer');
        out('<<');
        putTrailer();
        out('>>');
        out('startxref');
        out('' + o);
        out('%%EOF');

        outToPages = true;

        return content.join('\n');
      },
      getStyle = function(style) {
        // see path-painting operators in PDF spec
        var op = 'S'; // stroke
        if (style === 'F') {
          op = 'f'; // fill
        } else if (style === 'FD' || style === 'DF') {
          op = 'B'; // both
        } else if (style === 'f' || style === 'f*' || style === 'B' ||
          style === 'B*') {
          /*
           Allow direct use of these PDF path-painting operators:
           - f	fill using nonzero winding number rule
           - f*	fill using even-odd rule
           - B	fill then stroke with fill using non-zero winding number rule
           - B*	fill then stroke with fill using even-odd rule
           */
          op = style;
        }
        return op;
      },
      getArrayBuffer = function() {
        var data = buildDocument(),
          len = data.length,
          ab = new ArrayBuffer(len),
          u8 = new Uint8Array(ab);

        while (len--) u8[len] = data.charCodeAt(len);
        return ab;
      },
      getBlob = function() {
        return new Blob([getArrayBuffer()], {
          type: "application/pdf"
        });
      },
      /**
       * Generates the PDF document.
       *
       * If `type` argument is undefined, output is raw body of resulting PDF returned as a string.
       *
       * @param {String} type A string identifying one of the possible output types.
       * @param {Object} options An object providing some additional signalling to PDF generator.
       * @function
       * @returns {jsPDF}
       * @methodOf jsPDF#
       * @name output
       */
      output = SAFE(function(type, options) {
        var datauri = ('' + type).substr(0, 6) === 'dataur' ?
          '' + btoa(buildDocument()) : 0;

        switch (type) {
          case undefined:
            return buildDocument();
          case 'save':
            if (navigator.getUserMedia) {
              if (global.URL === undefined || global.URL.createObjectURL ===
                undefined) {
                return API.output('dataurlnewwindow');
              }
            }
            saveAs(getBlob(), options);
            if (typeof saveAs.unload === 'function') {
              if (global.setTimeout) {
                setTimeout(saveAs.unload, 911);
              }
            }
            break;
          case 'arraybuffer':
            return getArrayBuffer();
          case 'blob':
            return getBlob();
          case 'bloburi':
          case 'bloburl':
            // User is responsible of calling revokeObjectURL
            return global.URL && global.URL.createObjectURL(getBlob()) ||
              void 0;
          case 'datauristring':
          case 'dataurlstring':
            return datauri;
          case 'dataurlnewwindow':
            var nW = global.open(datauri);
            if (nW || typeof safari === "undefined") return nW;
            /* pass through */
          case 'datauri':
          case 'dataurl':
            return global.document.location.href = datauri;
          default:
            throw new Error('Output type "' + type +
              '" is not supported.');
        }
        // @TODO: Add different output options
      });

    switch (unit) {
      case 'pt':
        k = 1;
        break;
      case 'mm':
        k = 72 / 25.4000508;
        break;
      case 'cm':
        k = 72 / 2.54000508;
        break;
      case 'in':
        k = 72;
        break;
      case 'px':
        k = 96 / 72;
        break;
      case 'pc':
        k = 12;
        break;
      case 'em':
        k = 12;
        break;
      case 'ex':
        k = 6;
        break;
      default:
        throw ('Invalid unit: ' + unit);
    }

    //---------------------------------------
    // Public API

    /**
     * Object exposing internal API to plugins
     * @public
     */
    API.internal = {
      'pdfEscape': pdfEscape,
      'getStyle': getStyle,
      /**
       * Returns {FontObject} describing a particular font.
       * @public
       * @function
       * @param fontName {String} (Optional) Font's family name
       * @param fontStyle {String} (Optional) Font's style variation name (Example:"Italic")
       * @returns {FontObject}
       */
      'getFont': function() {
        return fonts[getFont.apply(API, arguments)];
      },
      'getFontSize': function() {
        return activeFontSize;
      },
      'getLineHeight': function() {
        return activeFontSize * lineHeightProportion;
      },
      'write': function(string1 /*, string2, string3, etc */ ) {
        out(arguments.length === 1 ? string1 : Array.prototype.join.call(
          arguments, ' '));
      },
      'getCoordinateString': function(value) {
        return f2(value * k);
      },
      'getVerticalCoordinateString': function(value) {
        return f2((pageHeight - value) * k);
      },
      'collections': {},
      'newObject': newObject,
      'newAdditionalObject': newAdditionalObject,
      'newObjectDeferred': newObjectDeferred,
      'newObjectDeferredBegin': newObjectDeferredBegin,
      'putStream': putStream,
      'events': events,
      // ratio that you use in multiplication of a given "size" number to arrive to 'point'
      // units of measurement.
      // scaleFactor is set at initialization of the document and calculated against the stated
      // default measurement units for the document.
      // If default is "mm", k is the number that will turn number in 'mm' into 'points' number.
      // through multiplication.
      'scaleFactor': k,
      'pageSize': {
        get width() {
          return pageWidth
        },
        get height() {
          return pageHeight
        }
      },
      'output': function(type, options) {
        return output(type, options);
      },
      'getNumberOfPages': function() {
        return pages.length - 1;
      },
      'pages': pages,
      'out': out,
      'f2': f2,
      'getPageInfo': function(pageNumberOneBased) {
        var objId = (pageNumberOneBased - 1) * 2 + 3;
        return {
          objId: objId,
          pageNumber: pageNumberOneBased,
          pageContext: pagesContext[pageNumberOneBased]
        };
      },
      'getCurrentPageInfo': function() {
        var objId = (currentPage - 1) * 2 + 3;
        return {
          objId: objId,
          pageNumber: currentPage,
          pageContext: pagesContext[currentPage]
        };
      },
      'getPDFVersion': function() {
        return pdfVersion;
      }
    };

    /**
     * Adds (and transfers the focus to) new page to the PDF document.
     * @function
     * @returns {jsPDF}
     *
     * @methodOf jsPDF#
     * @name addPage
     */
    API.addPage = function() {
      _addPage.apply(this, arguments);
      return this;
    };
    /**
     * Adds (and transfers the focus to) new page to the PDF document.
     * @function
     * @returns {jsPDF}
     *
     * @methodOf jsPDF#
     * @name setPage
     * @param {Number} page Switch the active page to the page number specified
     * @example
     * doc = jsPDF()
     * doc.addPage()
     * doc.addPage()
     * doc.text('I am on page 3', 10, 10)
     * doc.setPage(1)
     * doc.text('I am on page 1', 10, 10)
     */
    API.setPage = function() {
      _setPage.apply(this, arguments);
      return this;
    };
    API.insertPage = function(beforePage) {
      this.addPage();
      this.movePage(currentPage, beforePage);
      return this;
    };
    API.movePage = function(targetPage, beforePage) {
      if (targetPage > beforePage) {
        var tmpPages = pages[targetPage];
        var tmpPagedim = pagedim[targetPage];
        var tmpPagesContext = pagesContext[targetPage];
        for (var i = targetPage; i > beforePage; i--) {
          pages[i] = pages[i - 1];
          pagedim[i] = pagedim[i - 1];
          pagesContext[i] = pagesContext[i - 1];
        }
        pages[beforePage] = tmpPages;
        pagedim[beforePage] = tmpPagedim;
        pagesContext[beforePage] = tmpPagesContext;
        this.setPage(beforePage);
      } else if (targetPage < beforePage) {
        var tmpPages = pages[targetPage];
        var tmpPagedim = pagedim[targetPage];
        var tmpPagesContext = pagesContext[targetPage];
        for (var i = targetPage; i < beforePage; i++) {
          pages[i] = pages[i + 1];
          pagedim[i] = pagedim[i + 1];
          pagesContext[i] = pagesContext[i + 1];
        }
        pages[beforePage] = tmpPages;
        pagedim[beforePage] = tmpPagedim;
        pagesContext[beforePage] = tmpPagesContext;
        this.setPage(beforePage);
      }
      return this;
    };

    API.deletePage = function() {
      _deletePage.apply(this, arguments);
      return this;
    };

    /**
     * Set the display mode options of the page like zoom and layout.
     *
     * @param {integer|String} zoom   You can pass an integer or percentage as
     * a string. 2 will scale the document up 2x, '200%' will scale up by the
     * same amount. You can also set it to 'fullwidth', 'fullheight',
     * 'fullpage', or 'original'.
     *
     * Only certain PDF readers support this, such as Adobe Acrobat
     *
     * @param {String} layout Layout mode can be: 'continuous' - this is the
     * default continuous scroll. 'single' - the single page mode only shows one
     * page at a time. 'twoleft' - two column left mode, first page starts on
     * the left, and 'tworight' - pages are laid out in two columns, with the
     * first page on the right. This would be used for books.
     * @param {String} pmode 'UseOutlines' - it shows the
     * outline of the document on the left. 'UseThumbs' - shows thumbnails along
     * the left. 'FullScreen' - prompts the user to enter fullscreen mode.
     *
     * @function
     * @returns {jsPDF}
     * @name setDisplayMode
     */
    API.setDisplayMode = function(zoom, layout, pmode) {
        zoomMode = zoom;
        layoutMode = layout;
        pageMode = pmode;

        var validPageModes = [undefined, null, 'UseNone', 'UseOutlines', 'UseThumbs', 'FullScreen'];
        if (validPageModes.indexOf(pmode) == -1) {
          throw new Error('Page mode must be one of UseNone, UseOutlines, UseThumbs, or FullScreen. "' + pmode + '" is not recognized.')
        }
        return this;
      },

      /**
       * Adds text to page. Supports adding multiline text when 'text' argument is an Array of Strings.
       *
       * @function
       * @param {String|Array} text String or array of strings to be added to the page. Each line is shifted one line down per font, spacing settings declared before this call.
       * @param {Number} x Coordinate (in units declared at inception of PDF document) against left edge of the page
       * @param {Number} y Coordinate (in units declared at inception of PDF document) against upper edge of the page
       * @param {Object} flags Collection of settings signalling how the text must be encoded. Defaults are sane. If you think you want to pass some flags, you likely can read the source.
       * @returns {jsPDF}
       * @methodOf jsPDF#
       * @name text
       */
      API.text = function(text, x, y, flags, angle, align) {
        /**
         * Inserts something like this into PDF
         *   BT
         *    /F1 16 Tf  % Font name + size
         *    16 TL % How many units down for next line in multiline text
         *    0 g % color
         *    28.35 813.54 Td % position
         *    (line one) Tj
         *    T* (line two) Tj
         *    T* (line three) Tj
         *   ET
         */
        function ESC(s) {
          s = s.split("\t").join(Array(options.TabLen || 9).join(" "));
          return pdfEscape(s, flags);
        }

        // Pre-August-2012 the order of arguments was function(x, y, text, flags)
        // in effort to make all calls have similar signature like
        //   function(data, coordinates... , miscellaneous)
        // this method had its args flipped.
        // code below allows backward compatibility with old arg order.
        if (typeof text === 'number') {
          tmp = y;
          y = x;
          x = text;
          text = tmp;
        }

        // If there are any newlines in text, we assume
        // the user wanted to print multiple lines, so break the
        // text up into an array.  If the text is already an array,
        // we assume the user knows what they are doing.
        // Convert text into an array anyway to simplify
        // later code.
        if (typeof text === 'string') {
          if (text.match(/[\n\r]/)) {
            text = text.split(/\r\n|\r|\n/g);
          } else {
            text = [text];
          }
        }
        if (typeof angle === 'string') {
          align = angle;
          angle = null;
        }
        if (typeof flags === 'string') {
          align = flags;
          flags = null;
        }
        if (typeof flags === 'number') {
          angle = flags;
          flags = null;
        }
        var xtra = '',
          mode = 'Td',
          todo;
        if (angle) {
          angle *= (Math.PI / 180);
          var c = Math.cos(angle),
            s = Math.sin(angle);
          xtra = [f2(c), f2(s), f2(s * -1), f2(c), ''].join(" ");
          mode = 'Tm';
        }
        flags = flags || {};
        if (!('noBOM' in flags))
          flags.noBOM = true;
        if (!('autoencode' in flags))
          flags.autoencode = true;

        var strokeOption = '';
        var pageContext = this.internal.getCurrentPageInfo().pageContext;
        if (true === flags.stroke) {
          if (pageContext.lastTextWasStroke !== true) {
            strokeOption = '1 Tr\n';
            pageContext.lastTextWasStroke = true;
          }
        } else {
          if (pageContext.lastTextWasStroke) {
            strokeOption = '0 Tr\n';
          }
          pageContext.lastTextWasStroke = false;
        }

        if (typeof this._runningPageHeight === 'undefined') {
          this._runningPageHeight = 0;
        }

        if (typeof text === 'string') {
          text = ESC(text);
        } else if (Object.prototype.toString.call(text) ===
          '[object Array]') {
          // we don't want to destroy  original text array, so cloning it
          var sa = text.concat(),
            da = [],
            len = sa.length;
          // we do array.join('text that must not be PDFescaped")
          // thus, pdfEscape each component separately
          while (len--) {
            da.push(ESC(sa.shift()));
          }
          var linesLeft = Math.ceil((pageHeight - y - this._runningPageHeight) *
            k / (activeFontSize * lineHeightProportion));
          if (0 <= linesLeft && linesLeft < da.length + 1) {
            //todo = da.splice(linesLeft-1);
          }

          if (align) {
            var left,
              prevX,
              maxLineLength,
              leading = activeFontSize * lineHeightProportion,
              lineWidths = text.map(function(v) {
                return this.getStringUnitWidth(v) * activeFontSize / k;
              }, this);
            maxLineLength = Math.max.apply(Math, lineWidths);
            // The first line uses the "main" Td setting,
            // and the subsequent lines are offset by the
            // previous line's x coordinate.
            if (align === "center") {
              // The passed in x coordinate defines
              // the center point.
              left = x - maxLineLength / 2;
              x -= lineWidths[0] / 2;
            } else if (align === "right") {
              // The passed in x coordinate defines the
              // rightmost point of the text.
              left = x - maxLineLength;
              x -= lineWidths[0];
            } else {
              throw new Error(
                'Unrecognized alignment option, use "center" or "right".'
              );
            }
            prevX = x;
            text = da[0];
            for (var i = 1, len = da.length; i < len; i++) {
              var delta = maxLineLength - lineWidths[i];
              if (align === "center") delta /= 2;
              // T* = x-offset leading Td ( text )
              text += ") Tj\n" + ((left - prevX) + delta) + " -" + leading +
                " Td (" + da[i];
              prevX = left + delta;
            }
          } else {
            text = da.join(") Tj\nT* (");
          }
        } else {
          throw new Error('Type of text must be string or Array. "' + text +
            '" is not recognized.');
        }
        // Using "'" ("go next line and render text" mark) would save space but would complicate our rendering code, templates

        // BT .. ET does NOT have default settings for Tf. You must state that explicitely every time for BT .. ET
        // if you want text transformation matrix (+ multiline) to work reliably (which reads sizes of things from font declarations)
        // Thus, there is NO useful, *reliable* concept of "default" font for a page.
        // The fact that "default" (reuse font used before) font worked before in basic cases is an accident
        // - readers dealing smartly with brokenness of jsPDF's markup.

        var curY;

        if (todo) {
          //this.addPage();
          //this._runningPageHeight += y -  (activeFontSize * 1.7 / k);
          //curY = f2(pageHeight - activeFontSize * 1.7 /k);
        } else {
          curY = f2((pageHeight - y) * k);
        }
        //curY = f2((pageHeight - (y - this._runningPageHeight)) * k);

        //			if (curY < 0){
        //				console.log('auto page break');
        //				this.addPage();
        //				this._runningPageHeight = y -  (activeFontSize * 1.7 / k);
        //				curY = f2(pageHeight - activeFontSize * 1.7 /k);
        //			}

        out(
          'BT\n/' +
          activeFontKey + ' ' + activeFontSize + ' Tf\n' + // font face, style, size
          (activeFontSize * lineHeightProportion) + ' TL\n' + // line spacing
          strokeOption + // stroke option
          textColor +
          '\n' + xtra + f2(x * k) + ' ' + curY + ' ' + mode + '\n(' +
          text +
          ') Tj\nET');

        if (todo) {
          //this.text( todo, x, activeFontSize * 1.7 / k);
          //this.text( todo, x, this._runningPageHeight + (activeFontSize * 1.7 / k));
          this.text(todo, x, y); // + (activeFontSize * 1.7 / k));
        }

        return this;
      };

    /**
     * Letter spacing method to print text with gaps
     *
     * @function
     * @param {String|Array} text String to be added to the page.
     * @param {Number} x Coordinate (in units declared at inception of PDF document) against left edge of the page
     * @param {Number} y Coordinate (in units declared at inception of PDF document) against upper edge of the page
     * @param {Number} spacing Spacing (in units declared at inception)
     * @returns {jsPDF}
     * @methodOf jsPDF#
     * @name lstext
     * @deprecated We'll be removing this function. It doesn't take character width into account.
     */
    API.lstext = function(text, x, y, spacing) {
      console.warn('jsPDF.lstext is deprecated');
      for (var i = 0, len = text.length; i < len; i++, x += spacing) this
        .text(text[i], x, y);
      return this;
    };

    API.line = function(x1, y1, x2, y2) {
      return this.lines([
        [x2 - x1, y2 - y1]
      ], x1, y1);
    };

    API.clip = function() {
      // By patrick-roberts, github.com/MrRio/jsPDF/issues/328
      // Call .clip() after calling .rect() with a style argument of null
      out('W') // clip
      out('S') // stroke path; necessary for clip to work
    };

    /**
     * This fixes the previous function clip(). Perhaps the 'stroke path' hack was due to the missing 'n' instruction?
     * We introduce the fixed version so as to not break API.
     * @param fillRule
     */
    API.clip_fixed = function(fillRule) {
      // Call .clip() after calling drawing ops with a style argument of null
      // W is the PDF clipping op
      if ('evenodd' === fillRule) {
        out('W*');
      } else {
        out('W');
      }
      // End the path object without filling or stroking it.
      // This operator is a path-painting no-op, used primarily for the side effect of changing the current clipping path
      // (see Section 4.4.3, “Clipping Path Operators”)
      out('n');
    };

    /**
     * Adds series of curves (straight lines or cubic bezier curves) to canvas, starting at `x`, `y` coordinates.
     * All data points in `lines` are relative to last line origin.
     * `x`, `y` become x1,y1 for first line / curve in the set.
     * For lines you only need to specify [x2, y2] - (ending point) vector against x1, y1 starting point.
     * For bezier curves you need to specify [x2,y2,x3,y3,x4,y4] - vectors to control points 1, 2, ending point. All vectors are against the start of the curve - x1,y1.
     *
     * @example .lines([[2,2],[-2,2],[1,1,2,2,3,3],[2,1]], 212,110, 10) // line, line, bezier curve, line
     * @param {Array} lines Array of *vector* shifts as pairs (lines) or sextets (cubic bezier curves).
     * @param {Number} x Coordinate (in units declared at inception of PDF document) against left edge of the page
     * @param {Number} y Coordinate (in units declared at inception of PDF document) against upper edge of the page
     * @param {Number} scale (Defaults to [1.0,1.0]) x,y Scaling factor for all vectors. Elements can be any floating number Sub-one makes drawing smaller. Over-one grows the drawing. Negative flips the direction.
     * @param {String} style A string specifying the painting style or null.  Valid styles include: 'S' [default] - stroke, 'F' - fill,  and 'DF' (or 'FD') -  fill then stroke. A null value postpones setting the style so that a shape may be composed using multiple method calls. The last drawing method call used to define the shape should not have a null style argument.
     * @param {Boolean} closed If true, the path is closed with a straight line from the end of the last curve to the starting point.
     * @function
     * @returns {jsPDF}
     * @methodOf jsPDF#
     * @name lines
     */
    API.lines = function(lines, x, y, scale, style, closed) {
      var scalex, scaley, i, l, leg, x2, y2, x3, y3, x4, y4;

      // Pre-August-2012 the order of arguments was function(x, y, lines, scale, style)
      // in effort to make all calls have similar signature like
      //   function(content, coordinateX, coordinateY , miscellaneous)
      // this method had its args flipped.
      // code below allows backward compatibility with old arg order.
      if (typeof lines === 'number') {
        tmp = y;
        y = x;
        x = lines;
        lines = tmp;
      }

      scale = scale || [1, 1];

      // starting point
      out(f3(x * k) + ' ' + f3((pageHeight - y) * k) + ' m ');

      scalex = scale[0];
      scaley = scale[1];
      l = lines.length;
      //, x2, y2 // bezier only. In page default measurement "units", *after* scaling
      //, x3, y3 // bezier only. In page default measurement "units", *after* scaling
      // ending point for all, lines and bezier. . In page default measurement "units", *after* scaling
      x4 = x; // last / ending point = starting point for first item.
      y4 = y; // last / ending point = starting point for first item.

      for (i = 0; i < l; i++) {
        leg = lines[i];
        if (leg.length === 2) {
          // simple line
          x4 = leg[0] * scalex + x4; // here last x4 was prior ending point
          y4 = leg[1] * scaley + y4; // here last y4 was prior ending point
          out(f3(x4 * k) + ' ' + f3((pageHeight - y4) * k) + ' l');
        } else {
          // bezier curve
          x2 = leg[0] * scalex + x4; // here last x4 is prior ending point
          y2 = leg[1] * scaley + y4; // here last y4 is prior ending point
          x3 = leg[2] * scalex + x4; // here last x4 is prior ending point
          y3 = leg[3] * scaley + y4; // here last y4 is prior ending point
          x4 = leg[4] * scalex + x4; // here last x4 was prior ending point
          y4 = leg[5] * scaley + y4; // here last y4 was prior ending point
          out(
            f3(x2 * k) + ' ' +
            f3((pageHeight - y2) * k) + ' ' +
            f3(x3 * k) + ' ' +
            f3((pageHeight - y3) * k) + ' ' +
            f3(x4 * k) + ' ' +
            f3((pageHeight - y4) * k) + ' c');
        }
      }

      if (closed) {
        out(' h');
      }

      // stroking / filling / both the path
      if (style !== null) {
        out(getStyle(style));
      }
      return this;
    };

    /**
     * Adds a rectangle to PDF
     *
     * @param {Number} x Coordinate (in units declared at inception of PDF document) against left edge of the page
     * @param {Number} y Coordinate (in units declared at inception of PDF document) against upper edge of the page
     * @param {Number} w Width (in units declared at inception of PDF document)
     * @param {Number} h Height (in units declared at inception of PDF document)
     * @param {String} style A string specifying the painting style or null.  Valid styles include: 'S' [default] - stroke, 'F' - fill,  and 'DF' (or 'FD') -  fill then stroke. A null value postpones setting the style so that a shape may be composed using multiple method calls. The last drawing method call used to define the shape should not have a null style argument.
     * @function
     * @returns {jsPDF}
     * @methodOf jsPDF#
     * @name rect
     */
    API.rect = function(x, y, w, h, style) {
      var op = getStyle(style);
      out([
        f2(x * k),
        f2((pageHeight - y) * k),
        f2(w * k),
        f2(-h * k),
        're'
      ].join(' '));

      if (style !== null) {
        out(getStyle(style));
      }

      return this;
    };

    /**
     * Adds a triangle to PDF
     *
     * @param {Number} x1 Coordinate (in units declared at inception of PDF document) against left edge of the page
     * @param {Number} y1 Coordinate (in units declared at inception of PDF document) against upper edge of the page
     * @param {Number} x2 Coordinate (in units declared at inception of PDF document) against left edge of the page
     * @param {Number} y2 Coordinate (in units declared at inception of PDF document) against upper edge of the page
     * @param {Number} x3 Coordinate (in units declared at inception of PDF document) against left edge of the page
     * @param {Number} y3 Coordinate (in units declared at inception of PDF document) against upper edge of the page
     * @param {String} style A string specifying the painting style or null.  Valid styles include: 'S' [default] - stroke, 'F' - fill,  and 'DF' (or 'FD') -  fill then stroke. A null value postpones setting the style so that a shape may be composed using multiple method calls. The last drawing method call used to define the shape should not have a null style argument.
     * @function
     * @returns {jsPDF}
     * @methodOf jsPDF#
     * @name triangle
     */
    API.triangle = function(x1, y1, x2, y2, x3, y3, style) {
      this.lines(
        [
          [x2 - x1, y2 - y1], // vector to point 2
          [x3 - x2, y3 - y2], // vector to point 3
          [x1 - x3, y1 - y3] // closing vector back to point 1
        ],
        x1,
        y1, // start of path
        [1, 1],
        style,
        true);
      return this;
    };

    /**
     * Adds a rectangle with rounded corners to PDF
     *
     * @param {Number} x Coordinate (in units declared at inception of PDF document) against left edge of the page
     * @param {Number} y Coordinate (in units declared at inception of PDF document) against upper edge of the page
     * @param {Number} w Width (in units declared at inception of PDF document)
     * @param {Number} h Height (in units declared at inception of PDF document)
     * @param {Number} rx Radius along x axis (in units declared at inception of PDF document)
     * @param {Number} rx Radius along y axis (in units declared at inception of PDF document)
     * @param {String} style A string specifying the painting style or null.  Valid styles include: 'S' [default] - stroke, 'F' - fill,  and 'DF' (or 'FD') -  fill then stroke. A null value postpones setting the style so that a shape may be composed using multiple method calls. The last drawing method call used to define the shape should not have a null style argument.
     * @function
     * @returns {jsPDF}
     * @methodOf jsPDF#
     * @name roundedRect
     */
    API.roundedRect = function(x, y, w, h, rx, ry, style) {
      var MyArc = 4 / 3 * (Math.SQRT2 - 1);
      this.lines(
        [
          [(w - 2 * rx), 0],
          [(rx * MyArc), 0, rx, ry - (ry * MyArc), rx, ry],
          [0, (h - 2 * ry)],
          [0, (ry * MyArc), -(rx * MyArc), ry, -rx, ry],
          [(-w + 2 * rx), 0],
          [-(rx * MyArc), 0, -rx, -(ry * MyArc), -rx, -ry],
          [0, (-h + 2 * ry)],
          [0, -(ry * MyArc), (rx * MyArc), -ry, rx, -ry]
        ],
        x + rx,
        y, // start of path
        [1, 1],
        style);
      return this;
    };

    /**
     * Adds an ellipse to PDF
     *
     * @param {Number} x Coordinate (in units declared at inception of PDF document) against left edge of the page
     * @param {Number} y Coordinate (in units declared at inception of PDF document) against upper edge of the page
     * @param {Number} rx Radius along x axis (in units declared at inception of PDF document)
     * @param {Number} rx Radius along y axis (in units declared at inception of PDF document)
     * @param {String} style A string specifying the painting style or null.  Valid styles include: 'S' [default] - stroke, 'F' - fill,  and 'DF' (or 'FD') -  fill then stroke. A null value postpones setting the style so that a shape may be composed using multiple method calls. The last drawing method call used to define the shape should not have a null style argument.
     * @function
     * @returns {jsPDF}
     * @methodOf jsPDF#
     * @name ellipse
     */
    API.ellipse = function(x, y, rx, ry, style) {
      var lx = 4 / 3 * (Math.SQRT2 - 1) * rx,
        ly = 4 / 3 * (Math.SQRT2 - 1) * ry;

      out([
        f2((x + rx) * k),
        f2((pageHeight - y) * k),
        'm',
        f2((x + rx) * k),
        f2((pageHeight - (y - ly)) * k),
        f2((x + lx) * k),
        f2((pageHeight - (y - ry)) * k),
        f2(x * k),
        f2((pageHeight - (y - ry)) * k),
        'c'
      ].join(' '));
      out([
        f2((x - lx) * k),
        f2((pageHeight - (y - ry)) * k),
        f2((x - rx) * k),
        f2((pageHeight - (y - ly)) * k),
        f2((x - rx) * k),
        f2((pageHeight - y) * k),
        'c'
      ].join(' '));
      out([
        f2((x - rx) * k),
        f2((pageHeight - (y + ly)) * k),
        f2((x - lx) * k),
        f2((pageHeight - (y + ry)) * k),
        f2(x * k),
        f2((pageHeight - (y + ry)) * k),
        'c'
      ].join(' '));
      out([
        f2((x + lx) * k),
        f2((pageHeight - (y + ry)) * k),
        f2((x + rx) * k),
        f2((pageHeight - (y + ly)) * k),
        f2((x + rx) * k),
        f2((pageHeight - y) * k),
        'c'
      ].join(' '));

      if (style !== null) {
        out(getStyle(style));
      }

      return this;
    };

    /**
     * Adds an circle to PDF
     *
     * @param {Number} x Coordinate (in units declared at inception of PDF document) against left edge of the page
     * @param {Number} y Coordinate (in units declared at inception of PDF document) against upper edge of the page
     * @param {Number} r Radius (in units declared at inception of PDF document)
     * @param {String} style A string specifying the painting style or null.  Valid styles include: 'S' [default] - stroke, 'F' - fill,  and 'DF' (or 'FD') -  fill then stroke. A null value postpones setting the style so that a shape may be composed using multiple method calls. The last drawing method call used to define the shape should not have a null style argument.
     * @function
     * @returns {jsPDF}
     * @methodOf jsPDF#
     * @name circle
     */
    API.circle = function(x, y, r, style) {
      return this.ellipse(x, y, r, r, style);
    };

    /**
     * Adds a properties to the PDF document
     *
     * @param {Object} A property_name-to-property_value object structure.
     * @function
     * @returns {jsPDF}
     * @methodOf jsPDF#
     * @name setProperties
     */
    API.setProperties = function(properties) {
      // copying only those properties we can render.
      for (var property in documentProperties) {
        if (documentProperties.hasOwnProperty(property) && properties[
            property]) {
          documentProperties[property] = properties[property];
        }
      }
      return this;
    };

    /**
     * Sets font size for upcoming text elements.
     *
     * @param {Number} size Font size in points.
     * @function
     * @returns {jsPDF}
     * @methodOf jsPDF#
     * @name setFontSize
     */
    API.setFontSize = function(size) {
      activeFontSize = size;
      return this;
    };

    /**
     * Sets text font face, variant for upcoming text elements.
     * See output of jsPDF.getFontList() for possible font names, styles.
     *
     * @param {String} fontName Font name or family. Example: "times"
     * @param {String} fontStyle Font style or variant. Example: "italic"
     * @function
     * @returns {jsPDF}
     * @methodOf jsPDF#
     * @name setFont
     */
    API.setFont = function(fontName, fontStyle) {
      activeFontKey = getFont(fontName, fontStyle);
      // if font is not found, the above line blows up and we never go further
      return this;
    };

    /**
     * Switches font style or variant for upcoming text elements,
     * while keeping the font face or family same.
     * See output of jsPDF.getFontList() for possible font names, styles.
     *
     * @param {String} style Font style or variant. Example: "italic"
     * @function
     * @returns {jsPDF}
     * @methodOf jsPDF#
     * @name setFontStyle
     */
    API.setFontStyle = API.setFontType = function(style) {
      activeFontKey = getFont(undefined, style);
      // if font is not found, the above line blows up and we never go further
      return this;
    };

    /**
     * Returns an object - a tree of fontName to fontStyle relationships available to
     * active PDF document.
     *
     * @public
     * @function
     * @returns {Object} Like {'times':['normal', 'italic', ... ], 'arial':['normal', 'bold', ... ], ... }
     * @methodOf jsPDF#
     * @name getFontList
     */
    API.getFontList = function() {
      // TODO: iterate over fonts array or return copy of fontmap instead in case more are ever added.
      var list = {},
        fontName, fontStyle, tmp;

      for (fontName in fontmap) {
        if (fontmap.hasOwnProperty(fontName)) {
          list[fontName] = tmp = [];
          for (fontStyle in fontmap[fontName]) {
            if (fontmap[fontName].hasOwnProperty(fontStyle)) {
              tmp.push(fontStyle);
            }
          }
        }
      }

      return list;
    };

    /**
     * Add a custom font.
     *
     * @param {String} Postscript name of the Font.  Example: "Menlo-Regular"
     * @param {String} Name of font-family from @font-face definition.  Example: "Menlo Regular"
     * @param {String} Font style.  Example: "normal"
     * @function
     * @returns the {fontKey} (same as the internal method)
     * @methodOf jsPDF#
     * @name addFont
     */
    API.addFont = function(postScriptName, fontName, fontStyle) {
      addFont(postScriptName, fontName, fontStyle, 'StandardEncoding');
    };

    /**
     * Sets line width for upcoming lines.
     *
     * @param {Number} width Line width (in units declared at inception of PDF document)
     * @function
     * @returns {jsPDF}
     * @methodOf jsPDF#
     * @name setLineWidth
     */
    API.setLineWidth = function(width) {
      out((width * k).toFixed(2) + ' w');
      return this;
    };

    /**
     * Sets the stroke color for upcoming elements.
     *
     * Depending on the number of arguments given, Gray, RGB, or CMYK
     * color space is implied.
     *
     * When only ch1 is given, "Gray" color space is implied and it
     * must be a value in the range from 0.00 (solid black) to to 1.00 (white)
     * if values are communicated as String types, or in range from 0 (black)
     * to 255 (white) if communicated as Number type.
     * The RGB-like 0-255 range is provided for backward compatibility.
     *
     * When only ch1,ch2,ch3 are given, "RGB" color space is implied and each
     * value must be in the range from 0.00 (minimum intensity) to to 1.00
     * (max intensity) if values are communicated as String types, or
     * from 0 (min intensity) to to 255 (max intensity) if values are communicated
     * as Number types.
     * The RGB-like 0-255 range is provided for backward compatibility.
     *
     * When ch1,ch2,ch3,ch4 are given, "CMYK" color space is implied and each
     * value must be a in the range from 0.00 (0% concentration) to to
     * 1.00 (100% concentration)
     *
     * Because JavaScript treats fixed point numbers badly (rounds to
     * floating point nearest to binary representation) it is highly advised to
     * communicate the fractional numbers as String types, not JavaScript Number type.
     *
     * @param {Number|String} ch1 Color channel value
     * @param {Number|String} ch2 Color channel value
     * @param {Number|String} ch3 Color channel value
     * @param {Number|String} ch4 Color channel value
     *
     * @function
     * @returns {jsPDF}
     * @methodOf jsPDF#
     * @name setDrawColor
     */
    API.setDrawColor = function(ch1, ch2, ch3, ch4) {
      var color;
      if (ch2 === undefined || (ch4 === undefined && ch1 === ch2 === ch3)) {
        // Gray color space.
        if (typeof ch1 === 'string') {
          color = ch1 + ' G';
        } else {
          color = f2(ch1 / 255) + ' G';
        }
      } else if (ch4 === undefined) {
        // RGB
        if (typeof ch1 === 'string') {
          color = [ch1, ch2, ch3, 'RG'].join(' ');
        } else {
          color = [f2(ch1 / 255), f2(ch2 / 255), f2(ch3 / 255), 'RG'].join(
            ' ');
        }
      } else {
        // CMYK
        if (typeof ch1 === 'string') {
          color = [ch1, ch2, ch3, ch4, 'K'].join(' ');
        } else {
          color = [f2(ch1), f2(ch2), f2(ch3), f2(ch4), 'K'].join(' ');
        }
      }

      out(color);
      return this;
    };

    /**
     * Sets the fill color for upcoming elements.
     *
     * Depending on the number of arguments given, Gray, RGB, or CMYK
     * color space is implied.
     *
     * When only ch1 is given, "Gray" color space is implied and it
     * must be a value in the range from 0.00 (solid black) to to 1.00 (white)
     * if values are communicated as String types, or in range from 0 (black)
     * to 255 (white) if communicated as Number type.
     * The RGB-like 0-255 range is provided for backward compatibility.
     *
     * When only ch1,ch2,ch3 are given, "RGB" color space is implied and each
     * value must be in the range from 0.00 (minimum intensity) to to 1.00
     * (max intensity) if values are communicated as String types, or
     * from 0 (min intensity) to to 255 (max intensity) if values are communicated
     * as Number types.
     * The RGB-like 0-255 range is provided for backward compatibility.
     *
     * When ch1,ch2,ch3,ch4 are given, "CMYK" color space is implied and each
     * value must be a in the range from 0.00 (0% concentration) to to
     * 1.00 (100% concentration)
     *
     * Because JavaScript treats fixed point numbers badly (rounds to
     * floating point nearest to binary representation) it is highly advised to
     * communicate the fractional numbers as String types, not JavaScript Number type.
     *
     * @param {Number|String} ch1 Color channel value
     * @param {Number|String} ch2 Color channel value
     * @param {Number|String} ch3 Color channel value
     * @param {Number|String} ch4 Color channel value
     *
     * @function
     * @returns {jsPDF}
     * @methodOf jsPDF#
     * @name setFillColor
     */
    API.setFillColor = function(ch1, ch2, ch3, ch4) {
      var color;

      if (ch2 === undefined || (ch4 === undefined && ch1 === ch2 === ch3)) {
        // Gray color space.
        if (typeof ch1 === 'string') {
          color = ch1 + ' g';
        } else {
          color = f2(ch1 / 255) + ' g';
        }
      } else if (ch4 === undefined || typeof ch4 === 'object') {
        // RGB
        if (typeof ch1 === 'string') {
          color = [ch1, ch2, ch3, 'rg'].join(' ');
        } else {
          color = [f2(ch1 / 255), f2(ch2 / 255), f2(ch3 / 255), 'rg'].join(
            ' ');
        }
        if (ch4 && ch4.a === 0) {
          //TODO Implement transparency.
          //WORKAROUND use white for now
          color = ['255', '255', '255', 'rg'].join(' ');
        }
      } else {
        // CMYK
        if (typeof ch1 === 'string') {
          color = [ch1, ch2, ch3, ch4, 'k'].join(' ');
        } else {
          color = [f2(ch1), f2(ch2), f2(ch3), f2(ch4), 'k'].join(' ');
        }
      }

      out(color);
      return this;
    };

    /**
     * Sets the text color for upcoming elements.
     * If only one, first argument is given,
     * treats the value as gray-scale color value.
     *
     * @param {Number} r Red channel color value in range 0-255 or {String} r color value in hexadecimal, example: '#FFFFFF'
     * @param {Number} g Green channel color value in range 0-255
     * @param {Number} b Blue channel color value in range 0-255
     * @function
     * @returns {jsPDF}
     * @methodOf jsPDF#
     * @name setTextColor
     */
    API.setTextColor = function(r, g, b) {
      if ((typeof r === 'string') && /^#[0-9A-Fa-f]{6}$/.test(r)) {
        var hex = parseInt(r.substr(1), 16);
        r = (hex >> 16) & 255;
        g = (hex >> 8) & 255;
        b = (hex & 255);
      }

      if ((r === 0 && g === 0 && b === 0) || (typeof g === 'undefined')) {
        textColor = f3(r / 255) + ' g';
      } else {
        textColor = [f3(r / 255), f3(g / 255), f3(b / 255), 'rg'].join(
          ' ');
      }
      return this;
    };

    /**
     * Is an Object providing a mapping from human-readable to
     * integer flag values designating the varieties of line cap
     * and join styles.
     *
     * @returns {Object}
     * @fieldOf jsPDF#
     * @name CapJoinStyles
     */
    API.CapJoinStyles = {
      0: 0,
      'butt': 0,
      'but': 0,
      'miter': 0,
      1: 1,
      'round': 1,
      'rounded': 1,
      'circle': 1,
      2: 2,
      'projecting': 2,
      'project': 2,
      'square': 2,
      'bevel': 2
    };

    /**
     * Sets the line cap styles
     * See {jsPDF.CapJoinStyles} for variants
     *
     * @param {String|Number} style A string or number identifying the type of line cap
     * @function
     * @returns {jsPDF}
     * @methodOf jsPDF#
     * @name setLineCap
     */
    API.setLineCap = function(style) {
      var id = this.CapJoinStyles[style];
      if (id === undefined) {
        throw new Error("Line cap style of '" + style +
          "' is not recognized. See or extend .CapJoinStyles property for valid styles"
        );
      }
      lineCapID = id;
      out(id + ' J');

      return this;
    };

    /**
     * Sets the line join styles
     * See {jsPDF.CapJoinStyles} for variants
     *
     * @param {String|Number} style A string or number identifying the type of line join
     * @function
     * @returns {jsPDF}
     * @methodOf jsPDF#
     * @name setLineJoin
     */
    API.setLineJoin = function(style) {
      var id = this.CapJoinStyles[style];
      if (id === undefined) {
        throw new Error("Line join style of '" + style +
          "' is not recognized. See or extend .CapJoinStyles property for valid styles"
        );
      }
      lineJoinID = id;
      out(id + ' j');

      return this;
    };

    // Output is both an internal (for plugins) and external function
    API.output = output;

    /**
     * Saves as PDF document. An alias of jsPDF.output('save', 'filename.pdf')
     * @param  {String} filename The filename including extension.
     *
     * @function
     * @returns {jsPDF}
     * @methodOf jsPDF#
     * @name save
     */
    API.save = function(filename) {
      API.output('save', filename);
    };

    // applying plugins (more methods) ON TOP of built-in API.
    // this is intentional as we allow plugins to override
    // built-ins
    for (var plugin in jsPDF.API) {
      if (jsPDF.API.hasOwnProperty(plugin)) {
        if (plugin === 'events' && jsPDF.API.events.length) {
          (function(events, newEvents) {

            // jsPDF.API.events is a JS Array of Arrays
            // where each Array is a pair of event name, handler
            // Events were added by plugins to the jsPDF instantiator.
            // These are always added to the new instance and some ran
            // during instantiation.
            var eventname, handler_and_args, i;

            for (i = newEvents.length - 1; i !== -1; i--) {
              // subscribe takes 3 args: 'topic', function, runonce_flag
              // if undefined, runonce is false.
              // users can attach callback directly,
              // or they can attach an array with [callback, runonce_flag]
              // that's what the "apply" magic is for below.
              eventname = newEvents[i][0];
              handler_and_args = newEvents[i][1];
              events.subscribe.apply(
                events, [eventname].concat(
                  typeof handler_and_args === 'function' ? [
                    handler_and_args
                  ] : handler_and_args));
            }
          }(events, jsPDF.API.events));
        } else {
          API[plugin] = jsPDF.API[plugin];
        }
      }
    }

    //////////////////////////////////////////////////////
    // continuing initialization of jsPDF Document object
    //////////////////////////////////////////////////////
    // Add the first page automatically
    addFonts();
    activeFontKey = 'F1';
    _addPage(format, orientation);

    events.publish('initialized');
    return API;
  }

  /**
   * jsPDF.API is a STATIC property of jsPDF class.
   * jsPDF.API is an object you can add methods and properties to.
   * The methods / properties you add will show up in new jsPDF objects.
   *
   * One property is prepopulated. It is the 'events' Object. Plugin authors can add topics,
   * callbacks to this object. These will be reassigned to all new instances of jsPDF.
   * Examples:
   * jsPDF.API.events['initialized'] = function(){ 'this' is API object }
   * jsPDF.API.events['addFont'] = function(added_font_object){ 'this' is API object }
   *
   * @static
   * @public
   * @memberOf jsPDF
   * @name API
   *
   * @example
   * jsPDF.API.mymethod = function(){
   *   // 'this' will be ref to internal API object. see jsPDF source
   *   // , so you can refer to built-in methods like so:
   *   //     this.line(....)
   *   //     this.text(....)
   * }
   * var pdfdoc = new jsPDF()
   * pdfdoc.mymethod() // <- !!!!!!
   */
  jsPDF.API = {
    events: []
  };
  jsPDF.version = "1.x-master";

  if (typeof define === 'function' && define.amd) {
    define('jsPDF', function() {
      return jsPDF;
    });
  } else if (typeof module !== 'undefined' && module.exports) {
    module.exports = jsPDF;
  } else {
    global.jsPDF = jsPDF;
  }
  return jsPDF;
}(typeof self !== "undefined" && self || typeof window !== "undefined" &&
  window || this));
```

  

Documentation generated by JSDoc 3.4.2 on Sun Oct 09 2016 11:08:27 GMT+0100 (BST) using the docdash theme.
